# The potential of artificial intelligence in clinical trials

**DOI:** 10.1111/eci.70182

**Published:** 2026-02-27

**Authors:** Rahul Aggarwal, Deepak L. Bhatt

**Affiliations:** ^1^ Brigham and Women's Hospital Heart and Vascular Center Harvard Medical School Boston Massachusetts USA; ^2^ Massachussetts Institute of Technology Cambridge Massachusetts USA; ^3^ Mount Sinai Fuster Heart Hospital, Icahn School of Medicine at Mount Sinai New York New York USA

**Keywords:** artificial intelligence, clinical trials, digital technology

## Abstract

**Background:**

Clinical trials are an important part of evidence generation in medicine but remain burdened by escalating costs, inefficiencies and manual processes. Artificial intelligence (AI) has emerged as a promising approach to address these limitations by improving efficiency across the trial lifecycle.

**Methods:**

In this review, we examine emerging applications of AI across the clinical trial lifecycle. We highlight key examples demonstrating feasibility and potential impact.

**Results:**

AI‐based approaches show promise in optimizing trial design, improving recruitment, streamlining conduct and enhancing data interpretation. Despite the potential of AI in trials, challenges persist, including data quality, regulatory and privacy concerns, as well as infrastructure issues. Ethical use will require strong governance frameworks emphasizing transparency and human oversight. The success of these technologies will depend on their continuous validation and monitoring of these technologies.

**Conclusions:**

With appropriate validation, monitoring and governance, AI could enable a more efficient, cost‐saving and effective clinical trial landscape that accelerates discovery.

## Graphical abstract

Artificial intelligence has the potential to transform many areas of clinical trials.

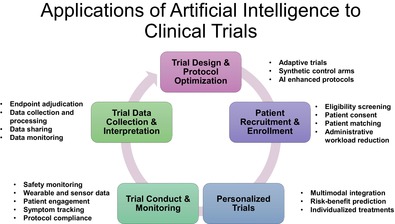

## 1 INTRODUCTION

Clinical trials remain the foundation of evidence generation in medicine and the gold standard for evaluating efficacy and safety of therapies, devices and care strategies. Yet, despite their important role, the traditional clinical trial enterprise is burdened with escalating costs, inefficiencies and operational challenges.^1^, ^2^ The cost of drug development can now exceed one billion dollars, with clinical trials being a significant part of these budgets.^3^, ^4^, ^5^, ^6^ Recruitment delays, high attrition rates and burdensome data collection processes further slow progress and can cause trials to be underpowered or infeasible.^7^, ^8^

Artificial intelligence (AI) represents an opportunity to rethink the clinical trial ecosystem.^9^, ^10^, ^11^ By leveraging advances in machine learning (ML), natural language processing (NLP) and generative AI (such as large language models [LLMs]), investigators can design more adaptive protocols, identify and engage eligible participants more effectively and streamline trial monitoring and endpoint assessment. Beyond automation, AI offers the potential to integrate large volumes of data and include multimodal capabilities.

**FIGURE 1:**
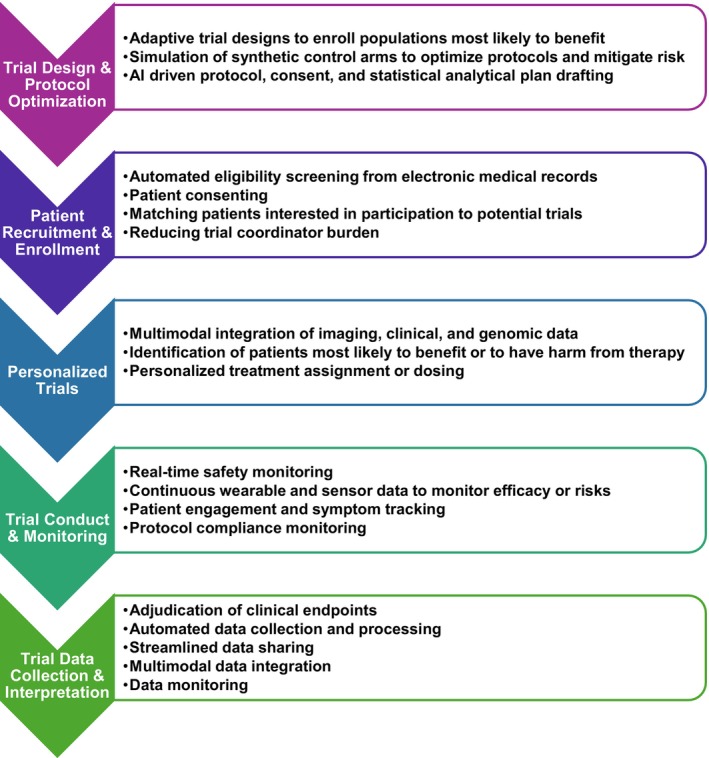
Artificial intelligence has the potential to transform multiple components of the clinical trial ecosystem. Key applications include optimizing trial design and protocol development, improving participant recruitment and enrollment, enhancing trial conduct and monitoring, and supporting data collection and interpretation.

In this review, we outline how AI technologies are reshaping the clinical trial lifecycle, including applications in trial design and protocol optimization, patient recruitment and enrollment, individualized risk prediction, trial conduct and monitoring, and data collection and interpretation (Figure 1). We focus on evidence‐based applications as well as promising upcoming approaches. We further explore the challenges inherent to AI implementation, and the ethical and regulatory considerations that will affect whether AI leads to more efficient, equitable and significant research.

## 2 APPLICATIONS OF AI TO CLINICAL TRIALS

### 2.1 Trial design and protocol optimization

Designing a clinical trial protocol is a key stage in the trial process, determining not only the validity of the scientific question but also the feasibility, safety and generalizability of the results. Traditional protocol development relies heavily on expert consensus and limited data, which can introduce inefficiencies, redundancies and avoidable trial failures.^12^, ^13^

ML now enables the large‐scale interrogation of historical trial datasets, real‐world evidence and electronic health records to inform protocol design.^14^, ^15^ These models can identify optimal inclusion and exclusion criteria, refine endpoint selection and predict operational feasibility—such as expected event rates or recruitment challenges—before a trial begins.^15^, ^16^ By modeling different design scenarios, AI can help anticipate risks and design protocols that are both scientifically rigorous and operationally efficient.^15^, ^17^

A rapidly emerging application is the development of AI simulations or synthetic control arms using ML, which can use historical patient data to project trial results in control arms before the trial starts.^18^, ^19^, ^20^, ^21^ This approach can reduce sample size requirements, mitigate risk and accelerate timelines.

Adaptive trial designs powered by ML are also a promising approach.^22^, ^23^, ^24^, ^25^, ^26^ Traditional trials are constrained by fixed enrollment criteria, which can lead to enrollment of low‐risk populations that can decrease the power and validity of a trial. ML‐based algorithms enable dynamic phenotyping, with continuous updating of inclusion criteria based on interim data, to enrich for patients most likely to benefit, mitigate recruitment issues and increase statistical power.^22^ Together, these advances can create a shift toward dynamic clinical trial systems, where protocols evolve intelligently in response to accruing evidence, ultimately closing the gap between research and real‐world care.

One such approach was described by Oikonomou and colleagues (Table 1).^22^ Using individual participant data from the Systolic Blood Pressure Intervention Trial (SPRINT) and Insulin Resistance Intervention after Stroke (IRIS) trials, the authors simulated an adaptive clinical trial framework guided by machine learning. Their algorithm determined phenotypic signatures of participants to predict individualized treatment benefit and then modified enrollment probabilities based on these predicted responses. Patients with a higher likelihood of benefit were preferentially weighted. This strategy, known as adaptive predictive enrichment, enabled a 14%–18% reduction in required sample size while maintaining comparable treatment effect estimates of the trials.^22^

**TABLE 1:** Examples of evidence for artificial intelligence benefit in clinical trials.

AI application	Example study	Study design	Results
Adaptive enrollment	Oikonomou et al., *NPJ Digit Med* 2023^22^	Used patient‐level data from SPRINT and IRIS to create machine‐learning derived phenotypes of patients. Used to simulate adaptive trial enrichment to prioritize high‐benefit participants in trials.	Reduced sample size necessary for the clinical trial by 14%–18%.
Eligibility screening	Unlu et al., *NEJM AI* 2024^29^	Applied an AI system to clinical notes to identify trial‐eligible heart‐failure patients and compared AI vs. manual screening.	Achieved 97%–100% accuracy of screening at a cost of ~$0.11 per patient.
Endpoint adjudication	Martí‐Castellote et al., *Circ Heart Fail* 2025^72^	Evaluated an NLP based system for heart failure hospitalization adjudication in DELIVER and compared performance to adjudication committee.	Demonstrated 91% agreement with experts and 84% reduction in workload after AI flagged uncertain events (16%) for manual review. Use of AI for adjudication led to similar treatment effect results (hazard ratio: 0.76 vs. 0.77).

Integrating AI into trial design with these approaches could reduce cost and duration, improve statistical power and accelerate trials. However, while the benefit is clear, it will be important to understand the impact of constantly changing enrollment criteria on the generalizability of the trials.

### 2.2 Patient recruitment and enrollment

Another bottleneck in clinical trials is the process of identifying, screening, enrolling and consenting patients.^25^ Traditional screening remains largely manual, requiring trained staff to review electronic health records (EHRs) to assess inclusion and exclusion criteria. This process is both resource‐intensive and error‐prone. While structured database queries can automate portions of the process, a large fraction of eligibility information resides in unstructured clinical notes that are difficult to evaluate at large scale.

AI with NLP and generative AI can enable automated, high‐accuracy patient screening from unstructured data.^27^, ^28^ An advantage is the ability to extract data from unstructured text. Unlu and colleagues showed an example of this with a LLM system termed RECTIFIER (Retrieval‐Augmented Generation–Enabled Clinical Trial Infrastructure for Inclusion–Exclusion Review), which was evaluated in the COPILOT‐HF trial (Table 1).^29^, ^30^ The system applied to clinical notes allowed automatic assessment of patient eligibility criteria that could not be determined through structured EHR data. RECTIFIER achieved 97.9%–100% accuracy across 13 eligibility criteria, exceeding that of trained human study staff (91.7%–100%), with particularly strong performance in identifying symptomatic heart failure (97.9% vs. 91.7% accuracy; MCC 0.924 vs. 0.721). Overall sensitivity and specificity for patient eligibility were 92.3% and 93.9%, respectively, compared with 90.1% and 83.6% for manual review. This system operated at an average cost of just $0.11 per patient, compared with an estimated $34.75 per patient for traditional phase 3 trial screening.^29^, ^31^

These findings demonstrate the potential of AI screening systems to reduce human burden, accelerate recruitment and reduce operational costs while maintaining or even improving accuracy. As clinical trial pipelines become more complex, AI screening systems could become foundational infrastructure, provided appropriate safeguards, clinician oversight and monitoring are in place.

### 2.3 Personalized trials

AI, and especially ML approaches, offer the potential for precision medicine through risk stratification and individualized treatment effect estimation.^32^, ^33^, ^34^, ^35^ Risk stratification can identify those most likely to experience an event, while predicting event rates, and individualized treatment effect estimation allows identifying those likely to have greater or less benefit from therapy. Rather than relying on population‐based averages, AI models can integrate imaging, clinical, biomarker, genetic and other complex data to estimate both an individual's risk for an outcome and differential response to specific therapies.^32^, ^36^, ^37^, ^38^, ^39^ This approach enables more targeted trial enrollment, preferentially including those that are most likely to benefit, and could reduce trial enrollment sample size.^34^, ^40^, ^41^

Precision medicine can allow for the design of creative trials with the goal of maximizing benefits of therapy and minimizing harms. One such example of its use in trials is the TRANSFORM trial.^42^ Prior evidence has shown that AI plaque analysis can improve cardiac risk stratification.^43^, ^44^, ^45^, ^46^, ^47^ In TRANSFORM, AI, specifically ML, is being used to determine individualized risk categories of coronary plaque based on coronary computed tomography angiography.^42^ Participants are being randomized to standard risk factor‐based care or to an AI‐guided group, where therapy intensity is tailored to individualized AI risk staging. By using AI‐based coronary staging, the trial aims to evaluate whether such an approach can improve personalized therapy choice by applying risk prediction to identify those more likely to benefit from intensive therapy with the goal of attenuating lipid‐rich plaque progression and reducing cardiovascular events compared with usual care.^42^ This approach optimizes trial therapy exposure based on projected potential benefit.

### 2.4 Trial conduct and monitoring

One of the challenges of clinical trials is capturing continuous data between study visits. Conventional data collection often involves visit‐based assessments that do not fully account for real‐time or changing data between visits. While the availability of remote monitoring technologies has grown tremendously, including home blood pressure monitors, digital weight scales and continuous glucose sensors, these devices generate large amounts of data that are challenging to interpret, integrate and operationalize into actionable insights.^48^, ^49^, ^50^, ^51^, ^52^

AI offers several mechanisms to improve between visit monitoring. First, AI‐enabled real‐time surveillance systems can continuously analyse incoming data to identify early safety signals, detect adverse events and trigger timely clinical interventions.^53^ Constant monitoring could enhances patient safety while potentially reducing the frequency of in‐person visits. Monitoring approaches could incorporate generative AI, ML, NLP or computer vision. Second, ML algorithms can synthesize vast multimodal data from wearables, laboratory systems and electronic health records to extract clinically meaningful trends and quantify treatment effects.^54^, ^55^, ^56^ These tools can detect subtle patterns or treatment responses that might be missed by conventional endpoints. Third, AI could be a means for increasing data intake and processing across different devices and platforms, significantly reducing site‐level data management burden.^57^

Beyond passive monitoring, AI could increasingly facilitate patient engagement between visits. Conversational agents, whether chatbot‐ or voice‐based, can guide medication adherence, collect patient‐reported outcomes and standardize symptom tracking in real time.^58^, ^59^ Early studies suggest that digital assistants can lead to user satisfaction and display empathy.^60^, ^61^, ^62^ These systems could further ensure protocol compliance and reduce trial attrition, though validation of these potential benefits requires further study.^63^, ^64^

Collectively, these innovations could demonstrate a shift toward proactive, continuous and patient‐centered trial operations, where AI not only enables real‐time decision support but also transforms the relationship between trial participants, investigators and data itself. While these benefits could be realized, understanding and monitoring the effects of AI implementation on human expertise and competency will also be necessary.

### 2.5 Trial data collection and interpretation

Possible benefits of AI include improving data collection and interpretation. AI could monitor for quality issues in trials.^65^ It could also consolidate fragmented data sources, extracting relevant events such as admissions for enrolled patients that occur outside of the trial site.^66^ Additionally, accurate endpoint determination is critical for clinical trial interpretation, yet the process remains a resource‐intensive and variable component of trial operations. Traditionally, adjudication committees manually review clinical records to confirm endpoints such as myocardial infarction or heart failure hospitalization, ensuring consistency and validity.^67^, ^68^ However, this approach is time‐consuming and expensive and the benefits are debated.^69^, ^70^, ^71^

AI provides an opportunity to improve this workflow. NLP tools and LLMs can rapidly extract and classify unstructured clinical data, converting free text into structured, analysable and useful information. Martí‐Castellote and colleagues evaluated this approach in the DELIVER trial, using an NLP adjudication model (Table 1).^72^ Among 1597 potential events, the AI system agreed with human adjudicators in 83% of cases and achieved 88% sensitivity. When used in a hybrid workflow that referred uncertain cases to human reviewers, accuracy increased to 91%, while manual effort was reduced by 84%. Critically, the estimated treatment effect of the study drug was nearly identical to traditional adjudication (hazard ratio 0.76 vs. 0.77).^72^

This approach shows that AI can reproduce expert‐level adjudication at a fraction of the cost and time while maintaining overall trial conclusions. As such systems improve, integrating them into trial workflows could enable faster, lower‐cost and more reproducible endpoint assessment—creating groundwork for real‐time, AI‐assisted outcome adjudication across trials. However, overdependence on automated adjudication could risk misdiagnosing clinically complex or context‐dependent cases that would otherwise be identified differently through expert human review.

## 3 KEY CONSIDERATIONS AND CHALLENGES

### 3.1 Data quality concerns

The performance and reliability of AI models in clinical research are dependent on the quality of the input data. Yet, real‐world clinical data often include inconsistent, incomplete or erroneous information (Table 2). In one large survey of over 22,000 patients with electronic health records, 21% reported errors in their medical notes, and among those, 42% perceived the errors as serious, relating to issues such as diagnostic details or medication lists.^73^ The effect of such data on accuracy of AI systems will be important to evaluate and monitor.

**TABLE 2:** Considerations and challenges of implementing artificial intelligence in clinical trials.

Area	Description	Potential mitigation strategies
Data Quality and Standardization	Clinical data incorrect, redundant or inconsistently formatted across sites and systems, leading to potential AI errors.	Establish standardized data pipelines, interoperability frameworks and regular data audits to ensure consistency and integrity.
Algorithmic Bias and Fairness	AI models may inadvertently reflect existing disparities if training data underrepresent certain populations or outcomes.	Use diverse, representative datasets, conduct bias audits, and incorporate fairness metrics into model evaluation. Validate models in diverse datasets.
Regulatory and Ethical Oversight	Current frameworks for regulating AI in research are evolving, with uncertainty around accountability, consent and model changes.	Align with emerging regulatory guidance, establish clear governance, version control and ethical review processes.
Data Privacy and Security	AI systems often require data sharing, increasing risks of breaches or unauthorized use.	Employ encryption, federated learning and strong data governance policies to protect participant confidentiality.
Infrastructure and Implementation	Variability in electronic health record systems and site capabilities complicate AI deployment and scalability.	Invest in interoperable infrastructure, cloud‐based analytics and centralized AI platforms to support multi‐site integration.

Data duplication and redundancy present an additional challenge. In an analysis of outpatient progress notes, Rule and colleagues found that median note redundancy was 59%, with 70% of text templated or copied from prior notes.^74^ This large volume of repeat data raises concern about AI's ability to extract important information being lost among repeat or unnecessary data. Further study to assess the impact of this on AI models will be necessary.

### 3.2 Regulatory and ethical considerations

The regulatory landscape will play an important role in how AI is applied within clinical trials. Although the U.S. Food and Drug Administration (FDA) has already authorized hundreds of AI‐enabled medical devices, regulatory frameworks for AI use within clinical research remain in early development.^75^ These policies are expected to evolve as regulators gain experience evaluating AI systems used for trials.

Regulatory pathways critical for the integration of AI into clinical research include the FDA's Medical Device Development Tools (MDDT) and Innovative Science and Technology Approaches for New Drugs (ISTAND) programs.^76^, ^77^ The MDDT program provides a mechanism to qualify AI tools used in the development of medical devices, such as digital biomarkers for measuring device performance.^76^ ISTAND focuses on drug development tools (DDTs).^77^ It is designed to evaluate novel technologies, including AI‐driven digital health tools, that do not fit traditional pathways. ISTAND can qualify AI algorithms intended for patient stratification, remote monitoring or endpoint assessment. Both programs aim to de‐risk regulatory acceptance for trial sponsors.

Central to oversight will be ensuring researchers can provide accountability and transparency, ensuring AI models have clearly documented audit trails, version controls and human‐in‐the‐loop supervision when necessary.^75^, ^78^, ^79^ Additionally, AI will need to be fair and carefully monitored to avoid exacerbating disparities, which could inadvertently cause bias in trial workflows.^80^, ^81^ One specific challenge for regulating and monitoring AI approaches is that often algorithms are difficult to interpret, as the mechanism by which the model has made predictions or decisions is unclear.^82^

Ethical oversight will require ensuring patient privacy, data integrity and overall safety of AI approaches.^83^, ^84^ Continuous monitoring of AI systems will be essential, especially as drift can cause model behaviour changes over time, which could lead to unforeseen AI output and safety risk.^75^, ^85^, ^86^, ^87^ Additionally, evaluation of whether AI can unblind clinical trial data will be necessary, a unique challenge that could arise if AI has access to large volumes of data.

### 3.3 Implementation challenges

Effective implementation of AI in clinical trials will depend heavily on data infrastructure, integration into existing workflows and interoperability.^88^, ^89^ One of the most immediate challenges is the infrastructure to deploy AI tools across multiple trial sites, each operating with separate EHR systems, software environments and data standards. Such fragmentation complicates both data harmonization and model deployment. Recent advances in data‐sharing frameworks have started to address these barriers. For instance, the 21st Century Cures Act has mandated patient access to their medical records, accelerating progress toward interoperability and standardized data exchange across healthcare systems.^90^ Additionally, the fast healthcare interoperability resources (FHIR) format has improved data standardization, though its utility for AI models needs further evaluation.^91^, ^92^ Decentralized trial models and centralized regulatory boards may help reduce some of the burdens of using AI in multisite trials.^93^, ^94^, ^95^

Data ownership, privacy and security remain concerns. Many AI‐enabled trial workflows require data to move across institutional or national boundaries, raising the risk of security breaches or unauthorized use.^96^, ^97^ Ensuring compliance with HIPAA and other local governance policies will be essential, along with employing encryption and approaches such as federated learning to protect participant information.^98^, ^99^ As AI adoption grows, strong infrastructure along with clear data stewardship frameworks will be critical to maintain trust, reproducibility and regulatory compliance in global clinical research.

## 4 CONCLUSIONS

AI has the potential to transform every stage of the clinical trial lifecycle—from design and recruitment to conduct, monitoring and data collection. AI has the ability to process vast, complex datasets, which can accelerate discovery, improve precision in patient selection and enhance the success of trials. Early evidence demonstrates that AI can streamline workflows, reduce costs and even match or surpass expert performance in key trial operations such as endpoint adjudication and patient screening. As adoption expands, these tools could enable more efficient, adaptive and patient‐centered trials that bring new therapies to patients faster and at lower costs.

Realizing the promise of AI in trials will require deliberate attention to data quality, equity, transparency and regulatory oversight. AI systems must be rigorously validated, continuously monitored, and integrated under robust governance frameworks that prioritize accountability, fairness and safety. Collaboration among clinicians, data scientists, regulators and industry will be essential to develop shared standards for AI use. Ultimately, the success of AI in clinical trials will depend not only on technological capabilities but also on the commitment to apply it ethically, ensuring that innovation enhances, rather than harms, the rigor and trust in clinical trials.

## FUNDING INFORMATION

Dr. Aggarwal receives research training support from the National Heart, Lung, and Blood Institute grant 5T32HL007604.

## CONFLICT OF INTEREST STATEMENT

There are no conflicts of interest related to the study design or its results. Outside of the manuscript: Dr. Aggarwal is involved in research funded by the Bristol Myers Squibb‐Pfizer alliance, Novartis, Lexicon, Cleerly and Amarin, is a consultant for Lexicon and Amarin, and has served on an advisory board for Bayer. Dr. Bhatt discloses the following relationships—Advisory Board: Angiowave, Antlia Bioscience, Bayer, Boehringer Ingelheim, CellProthera, Cereno Scientific, E‐Star Biotech, High Enrol, Janssen, Level Ex, McKinsey, Medscape Cardiology, Merck, NirvaMed, Novo Nordisk, Repair Biotechnologies, Stasys, SandboxAQ (stock options), Tourmaline Bio, Viatris; Board of Directors: American Heart Association New York City, Angiowave (stock options), Bristol Myers Squibb (stock), DRS.LINQ (stock options), High Enrol (stock); Consultant: Alnylam, Altimmune, Broadview Ventures, Corcept Therapeutics, Corsera, GlaxoSmithKline, Hims, SERB, SFJ, Summa Therapeutics, Worldwide Clinical Trials; Data Monitoring Committees: Acesion Pharma, Assistance Publique‐Hôpitaux de Paris, Baim Institute for Clinical Research, Boston Scientific (Chair, PEITHO trial), Cleveland Clinic, Contego Medical (Chair, PERFORMANCE 2), Duke Clinical Research Institute, Mayo Clinic, Mount Sinai School of Medicine (for the ABILITY‐DM trial, funded by Concept Medical; for ALLAY‐HF, funded by Alleviant Medical), Novartis, Population Health Research Institute; Rutgers University (for the NIH‐funded MINT Trial); Honoraria: American College of Cardiology (Senior Associate Editor, Clinical Trials and News, ACC.org; Chair, ACC Accreditation Oversight Committee), Arnold and Porter law firm (work related to Sanofi/Bristol‐Myers Squibb clopidogrel litigation), Baim Institute for Clinical Research (AEGIS‐II executive committee funded by CSL Behring), Belvoir Publications (Editor in Chief, Harvard Heart Letter), Canadian Medical and Surgical Knowledge Translation Research Group (clinical trial steering committees), CSL Behring (AHA lecture), Duke Clinical Research Institute, Engage Health Media, HMP Global (Editor in Chief, Journal of Invasive Cardiology), Medtelligence/ReachMD (CME steering committees), MJH Life Sciences, Oakstone CME (Course Director, Comprehensive Review of Interventional Cardiology), Philips (Becker's Webinar on AI), Population Health Research Institute, WebMD (CME steering committees), Wiley (steering committee); Other: Clinical Cardiology (Deputy Editor, unpaid); Progress in Cardiovascular Diseases (Deputy Editor, unpaid); Added Health (Editorial Board; stock options); Patent: Sotagliflozin (named on a patent for sotagliflozin assigned to Brigham and Women's Hospital who assigned to Lexicon; neither I nor Brigham and Women's Hospital receive any income from this patent); Research Funding: Abbott, Acesion Pharma, Afimmune, Alnylam, Amarin, Amgen, AstraZeneca, Atricure, Bayer, Boehringer Ingelheim, Boston Scientific, CellProthera, Cereno Scientific, Chiesi, Cleerly, CSL Behring, Faraday Pharmaceuticals, Fractyl, Idorsia, Janssen, Javelin, Lexicon, Lilly, Medtronic, Merck, MiRUS, Moderna, Novartis, Novo Nordisk, Pfizer, PhaseBio, Regeneron, Reid Hoffman Foundation, Roche, Sanofi, Stasys, 89Bio; Royalties: Elsevier (Editor, Braunwald's Heart Disease); Site Co‐Investigator: Cleerly.

## Data Availability

Data sharing not applicable as no new data were generated for this study.
